# Assessment of occupational risks in a fuel analysis laboratory

**DOI:** 10.47626/1679-4435-2026-1541

**Published:** 2026-09-27

**Authors:** Ana Claudia Almagro Alves de Souza, Debora Tomita Watanabe

**Affiliations:** 1 Faculdade de Ciências Médicas da Santa Casa, Departamento de Saúde Coletiva do Centro de Estudos Augusto Leopoldo Ayrosa Galvão, São Paulo, SP, Brazil

**Keywords:** fossil fuels, occupational hazards, safety management, occupational health

## Abstract

**Introduction:**

Occupational exposure to fuels and their chemical components represents a significant challenge to workers’ health, particularly in laboratory settings where these substances are handled directly and frequently. Although the toxic effects of these compounds have been widely described in the literature, studies systematically assessing the health profile of workers in fuel analysis laboratories remain scarce, particularly in Brazil.

**Objectives:**

To analyze occupational risks in a fuel analysis laboratory in the city of São Paulo by describing the workers’ health profile, the occurrence of signs and symptoms, and their possible relationship with chemical exposures, as well as to discuss the implications of the findings for improving preventive management practices in compliance with current regulatory standards.

**Methods:**

This was a cross-sectional, observational, descriptive study with a quantitative approach. A customized questionnaire, a customized Risk Recognition Form, an informed consent form, and the Advanced REACH Tool, version 1.5, were used.

**Results:**

The estimates obtained using the ART 1.5 tool, even under conservative assumptions, remained below the limits established by the American Conference of Governmental Industrial Hygienists and the values reported in an institutional document.

**Conclusions:**

Under the modeled conditions, the exposures estimated using the Advanced REACH Tool 1.5 remained below the established occupational exposure limits. Because this is a predictive tool, its results do not replace direct environmental monitoring. The study also identified opportunities to improve risk management and the laboratory infrastructure.

## INTRODUCTION

The Brazilian National Agency of Petroleum, Natural Gas and Biofuels is responsible for ensuring that fuels sold in Brazil meet quality standards by collecting samples from fuel retail stations and conducting analyses in specialized laboratories [^1^]. The main fuels subject to inspection include gasoline, ethanol, and diesel fuel.

Gasoline is a complex mixture of petroleum-derived hydrocarbons containing volatile aromatic compounds, such as benzene, toluene, and xylene, which are of recognized toxicological and occupational relevance [^1^-^3^]. Benzene is a volatile aromatic hydrocarbon widely distributed in the environment. However, occupational exposure is of greater toxicological relevance because of the intensity and chronicity of contact with this compound [^2^,^4^]. Benzene is classified as carcinogenic to humans (Group 1) by the International Agency for Research on Cancer, and no safe exposure threshold has been established [^5^,^6^]. In Brazil, its maximum permitted concentration in gasoline is 1% by volume [^7^,^8^]. Its acute effects include neurological and irritative manifestations [^5^,^6^].

Toluene is an aromatic hydrocarbon widely used in the chemical industry and found in automotive fuels [^4^,^9^]. Occupational exposure occurs through the inhalation of vapors or contact with the substance in liquid form and may cause acute neurotoxic effects and respiratory and skin irritation [^9^].

Commercial xylene is a mixture of isomers used as a solvent and may be present in small amounts in automotive fuels [^10^]. Occupational exposure may cause nonspecific neurological and irritative manifestations. More intense or chronic exposures may result in more severe hematological and cardiovascular abnormalities [^7^,^11^].

Ethanol is a biofuel widely used in Brazil as an alternative to gasoline [^12^]. Its regulation establishes limits for contaminants, including methanol [^12^]. Methanol is a highly toxic alcohol that may cause irritative manifestations and acute systemic symptoms following vapor inhalation [^13^].

Diesel fuel, which is derived from petroleum and composed of hydrocarbons, is used in transportation and power generation. Sulfur content is an important regulatory parameter associated with the control of atmospheric emissions [^14^]. Since 2005, biodiesel has been progressively added to commercial diesel fuel in Brazil, with the current proportion set at 15% [^15^]. Biodiesel, a renewable fuel obtained from vegetable oils or animal fats, helps reduce environmental impact compared with conventional fossil fuels [^15^].

In the context of occupational health and safety, a hazard is a source or situation with the potential to cause harm, whereas risk refers to the combination of the likelihood of that harm occurring and its severity [^16^]. This distinction guides the adoption of measures aimed at eliminating, preventing, or controlling occupational exposures.

Risk management encompasses measures related to the adequacy and maintenance of the work environment and resources, workplace layout, work organization, training, performance of activities, and worker protection, as established by the Brazilian Regulatory Standards (NRs) [^17^,^18^].

Hazard identification should consider the physicochemical properties of the substances used, their frequency and manner of use, the process in which they are used, the routes and sites of contact with the body, and the possibility of absorption, among other information [^19^]. Based on this identification, a quantitative assessment should be performed when indicated [^19^].

For chemical substances, occupational exposure limits (OELs) are technical reference values used to assess exposure. Although they do not guarantee the absence of adverse effects, in general, the lower the environmental concentration relative to the corresponding OEL, the lower the likelihood of harm tends to be [^19^].

Occupational exposure to fuels and their chemical components represents a significant challenge to workers’ health, particularly in laboratories where these substances are handled directly and frequently. Although the toxic effects of these compounds have been widely described in the literature, studies systematically assessing the health profile of workers in fuel analysis laboratories remain scarce, particularly in Brazil.

Because of the cross-sectional, observational, descriptive design of this study, the exploratory hypothesis was that occupational exposure to chemical agents in this environment could coexist with clinical manifestations consistent with the toxic effects of these agents. The aim was not to establish a causal relationship but to investigate the potential implications of this co-occurrence for occupational health surveillance and management.

Therefore, this study aimed to assess occupational risks in a fuel analysis laboratory in the city of São Paulo by describing the workers’ health profile, the occurrence of signs and symptoms, and the possible co-occurrence of these manifestations with chemical exposures as well as to identify opportunities to improve preventive management practices in compliance with current regulatory standards.

## METHODS

This was a cross-sectional, observational, and descriptive study with a quantitative approach, conducted between November 2023 and July 2024 in a fuel analysis laboratory located in the city of São Paulo. The study was exploratory and aimed to characterize occupational exposures, reported signs and symptoms, and existing management instruments, without seeking to establish causal relationships between exposures and health outcomes.

The study included 12 workers who voluntarily agreed to participate by signing the Informed Consent Form. The participants worked in the administrative, quality control, and laboratory analysis departments. Laboratory activities were organized according to the type of fuel analyzed (gasoline, ethanol, or diesel fuel) and performed on a rotational basis, with workers changing activities approximately every 3 months.

Workers of both sexes who were older than 18 years and performed their duties exclusively in the laboratory were included. Individuals who did not perform the activities described, those younger than 18 years, and workers who were on vacation or medical leave during the data collection period were excluded.

Data collection occurred in two stages. In the first stage, the risk recognition form, which addressed aspects related to the work environment, occupational safety, and occupational medicine, was completed. The Risk Management Program (PGR) and the Occupational Health Medical Control Program (PCMSO), which were made available by the company for consultation, were also reviewed. In the second stage, the production process and work organization were directly observed, and the workers were interviewed using a questionnaire developed by the researchers.

The questionnaire comprised 24 questions addressing sociodemographic and occupational characteristics, signs and symptoms experienced during working hours, absences from work, participation in educational programs, occupational safety training, work breaks, and meals.

The risk recognition form contained 82 questions addressing general company information, sanitary conditions, the structure and activities of the Specialized Service in Safety Engineering and Occupational Medicine, occupational hygiene and safety activities, occupational medicine, medical care, industrial waste, production process flow, departments, and workers’ roles.

During the first stage, the risk recognition form was completed at the company’s occupational health clinic. The physician in charge and the occupational safety engineer were interviewed to clarify the protocols and ensure that the data were completed appropriately.

The documentary review of the PGR and PCMSO was conducted through supervised access to the documents available on a company computer. The files were not made fully available to the researchers for extraction, copying, or subsequent independent review. Therefore, the documentary assessment was partial and restricted to the information accessible during the in-person consultations. To systematize the analysis, predefined criteria from the risk recognition form were adopted, including the identification of the chemical agents mentioned in the documents, the presence or absence of quantitative characterization, the correspondence between the risks identified in the PGR and the examinations specified in the PCMSO, and the consistency between environmental hazard identification and occupational health surveillance measures.

In the second stage, the questionnaire was administered to the workers, and a workplace visit was conducted to directly observe the production process and work organization. During the visit, the workers provided clarification regarding the analytical procedures performed, the equipment available, the main substances used, the division of the workplace into specific areas, cleaning and waste disposal procedures, and the flow of laboratory processes.

The Advanced REACH Tool, version 1.5 (ART 1.5), a predictive model designed to estimate occupational exposure through inhalation, was used. The model is based on information from databases on exposure variability, the available literature, and exposure measurements performed in specific settings [^20^,^21^]. Exposure to acetone, toluene, xylene, benzene, methanol, gasoline, ethanol, and diesel fuel was assessed based on the information provided in the available Safety Data Sheets for Chemical Products (FISPQs).

Because it is a predictive tool, the results of ART 1.5 are subject to uncertainties inherent in the model and depend on the quality, completeness, and representativeness of the information entered. This information included the physicochemical characteristics of the substances, the quantities handled, the duration and frequency of the activities, ventilation conditions, control measures, and the assumptions adopted to characterize the exposure scenarios. Therefore, the estimates obtained do not replace direct environmental monitoring but constitute a complementary tool for screening, preliminary characterization, and prioritization of situations requiring quantitative workplace assessment.

The study was considered to involve minimal risk. Participation was voluntary, and participants could withdraw their consent at any time without penalty.

## RESULTS

In the division of labor observed in the chemical analysis laboratory, four main job positions were identified: researcher, technician, assistant, and administrative assistant. Researchers, technicians, and assistants performed similar duties involving chemical analyses of fuel samples and the handling of pure substances (Figure 1). These positions differed according to the hierarchy associated with career progression. The administrative assistant was responsible for customer service, preparing quotations, organizing documents, and performing other administrative activities.

**Figure 1.**
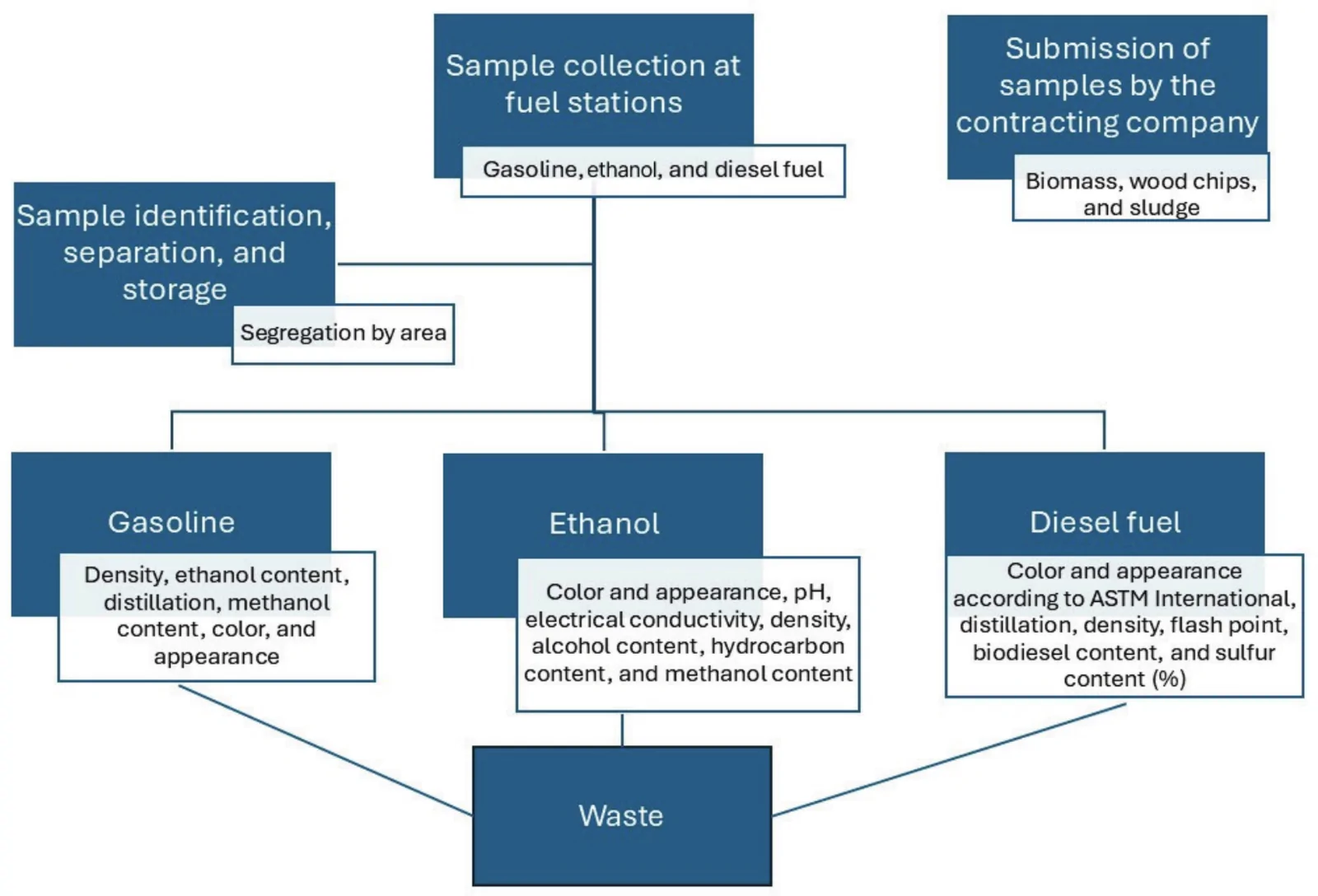
Workflow diagram. ASTM = American Society for Testing and Materials.

Source: Prepared by the authors.

The fuel samples used in the chemical analyses were collected by specialized agents at previously selected fuel stations. Regardless of the type of fuel (gasoline, ethanol, or diesel fuel), the samples were identified, separated, and stored. The main tests performed are described below:

- Distillation
  In this test, a distillation apparatus is used to quantitatively determine the boiling range of the products evaluated. A 100-mL sample is collected using a pipette. The sample is transferred to a graduated cylinder, in which its volume is precisely measured, and subsequently to a round-bottom flask connected to the distillation apparatus for the distillation process.
- Color and appearance
  In this visual test, the sample is transferred to a graduated cylinder, and its color and appearance, homogeneity, and the presence of particulate matter are assessed. A 1-L sample of gasoline, ethanol, or diesel fuel is used.
- pH
  This test is performed on ethanol samples. A pH meter and a 50-mL sample are used to determine the pH. Lithium chloride, sodium hydroxide, and hydrochloric acid are used in the operation and maintenance of the equipment.
- Biodiesel content
  A 50-mL sample of diesel fuel is transferred to a beaker and subsequently to a syringe. The analysis is performed using an infrared spectrometer connected to a computer to determine the biodiesel content.
- Electrical conductivity
  A conductivity meter and a 50-mL sample, generally of ethanol, are used. Hydrochloric acid, diethyl ether, and isopropyl alcohol are used in the operation and maintenance of the equipment.
- Ethanol content
  This test determines anhydrous ethanol content starting at 1% by volume in gasoline samples. To perform the test, 50 mL of the sample is transferred to a graduated cylinder, and the volume is brought to 100 mL with a 10% NaCl solution.
- Hydrocarbon content
  This test is performed to detect possible gasoline contamination. The procedure is similar to that used to determine ethanol content.
- Methanol content
  A 1-2-mL aliquot of the ethanol sample is injected into a porous capillary column in a gas chromatograph. The different components in the sample are separated by the column and subsequently detected by ionization.
- Density
  The density and relative density of gasoline, diesel fuel, ethanol, and lubricating oil samples are determined using a digital density meter. A 1-2-mL aliquot of the sample is used.
- Flash point
  The flash point is the lowest temperature at which the vapors of a substance ignite momentarily upon contact with an ignition source. Combustion ceases after the source is removed. A 100-mL sample is used. This property is used to assess the flammability risk of chemical substances and establish safety and transportation requirements.
- Sulfur
  Sulfur content is determined using a total sulfur analyzer based on ultraviolet fluorescence. A 5-mL sample, such as gasoline, is injected into a pyrolysis tube maintained at a temperature above 1,000 °C, in which sulfur is oxidized to sulfur dioxide (SO_2_). The solvents toluene, xylene, and isooctane are used in the operation and cleaning of the equipment.

Of the 12 workers interviewed, seven (58.3%) were men and five (41.7%) were women. The mean age was 43.3 years, ranging from 25 to 68 years. The mean length of employment at the company was 25.8 years, ranging from 1.5 to 45 years.

Considering all symptoms reported in the questionnaire, the most frequent were headache (26.6%) and rhinorrhea (20.0%), followed by nasal congestion (16.6%), fatigue (13.3%), dizziness (10.0%), eye irritation (6.7%), and drowsiness (6.7%). None of the participants reported chest pain, epistaxis, or anemia.

During the visit, the laboratory was clean and well lit, with artificial fluorescent lighting and natural light from closed rectangular windows located near the ceiling. The space was divided into areas designated for sample identification and storage, chemical analyses, waste disposal, and equipment cleaning (Figure 2). Four exhaust systems were installed above the workbenches, and three fume hoods were available for handling chemical compounds. An emergency shower and an eyewash station were readily accessible in the event of chemical accidents.

**Figure 2.**
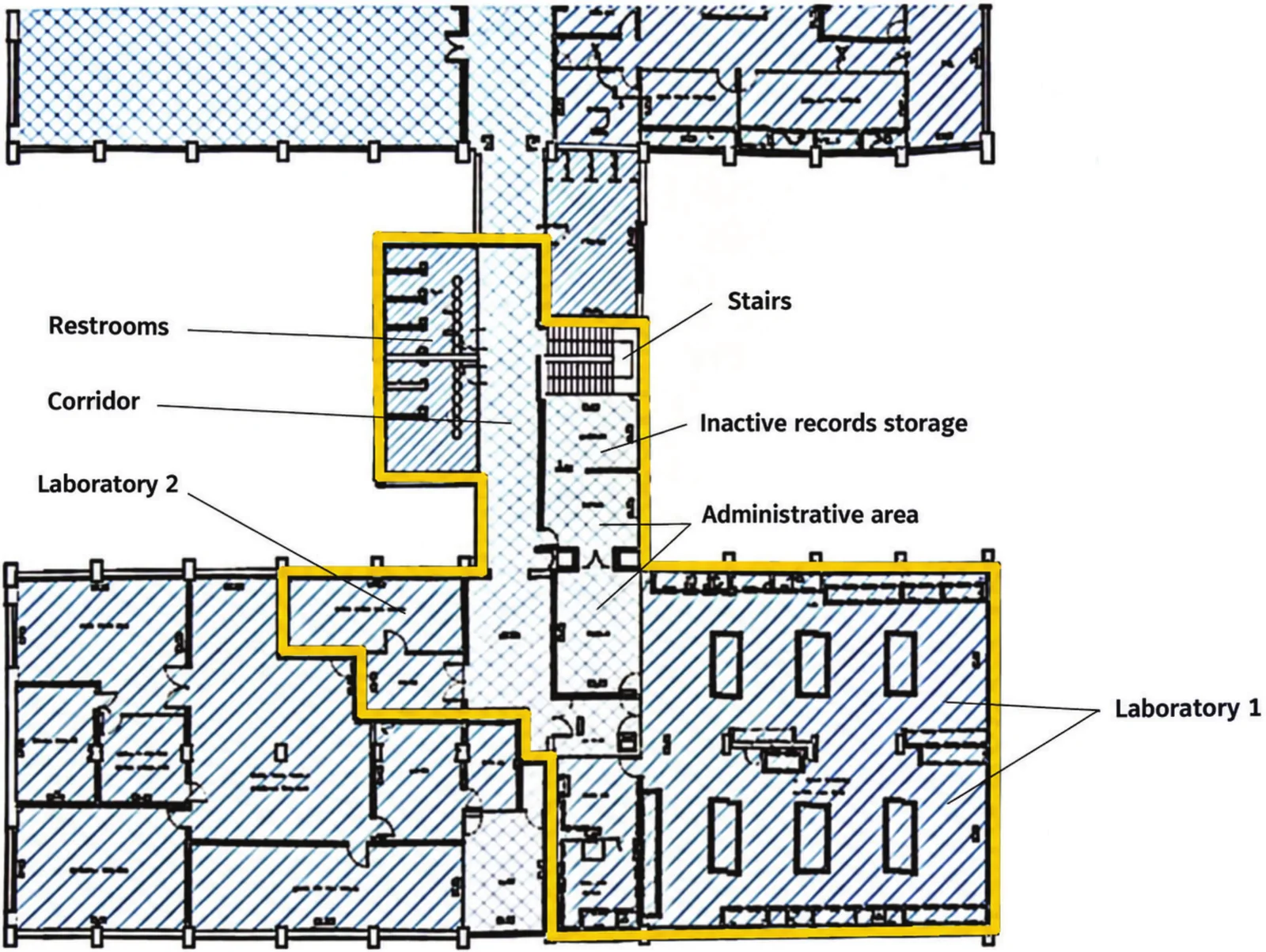
Floor plan of the fuel analysis laboratory.

Source: Prepared by the authors.

Fire extinguishers, hoses, and hydrants were readily accessible for firefighting. The distillation equipment was equipped with a manually activated fire suppression system that released a jet of carbon dioxide (CO_2_) directly onto the source of the fire. This system was installed because flammable fuels were heated during the distillation process, resulting in a risk of ignition. An emergency exit was also available. However, neither escape route signage nor an emergency floor plan providing guidance in the event of an incident was observed. Three doors that could be used for evacuation were identified, one of which remained locked by an electronic lock. The laboratory did not have a fire-rated door and was located on the second floor of the building.

An area separated from the laboratory by walls with glass windows and a wooden door was used to prepare documents, reports, and spreadsheets; provide customer service; organize schedules; and perform other administrative activities. This area contained desks and chairs for the workers. There was also a small room separated from the laboratory by a wall with windows and a wooden door that was used as a break room.

Across a corridor, in an area enclosed by walls and windows, there was a small annex to the main laboratory intended for diesel fuel analysis and sulfur content determination. This space had no exhaust systems or fume hoods and contained small workbenches, a sink, and specialized equipment. It was accessed through a single door. For the purposes of this study, the annex was designated laboratory 2 and the main laboratory was designated laboratory 1.

The waste generated in the laboratories was stored in appropriate containers and collected by a specialized company contracted to ensure its proper disposal. The waste was predominantly liquid and included residual fuel samples from the analyses and solvents, mainly acetone and toluene.

The occupational health clinic was located in another building and had a team comprising an occupational health nurse, a nursing technician, a supervising physician, and seven examining physicians, who worked in shifts and according to schedules from Monday through Friday. The PCMSO was prepared externally by a contracted medical company and signed by the occupational physician in charge. The clinic conducted periodic examinations, provided medical care and first aid, and could transfer workers to referral hospitals using its own ambulance or a company vehicle. Health care was provided through the company’s health insurance plan and extended to the workers’ family members.

In urgent or emergency cases, initial care was provided by the clinic staff, followed, when necessary, by transfer to a referral service. First-aid supplies complied with NR-7, and the available medications were intended for oral administration. No automated external defibrillator was available on the company premises. The Internal Commission for Accident and Harassment Prevention provided training on the urgent and emergency care program through lectures.

For the application of ART 1.5, the fuels gasoline, ethanol, and diesel fuel were selected, along with the three pure substances most frequently used in analyses and equipment cleaning: xylene, toluene, and acetone. Their respective FISPQs were examined to supplement the characterization of the substances.

Based on data obtained during the laboratory visit and the characterization of the work process, ART 1.5 could be applied. The tool performs predictive modeling of exposure to each selected substance or fuel based on data on exposure variability and the available literature. The results obtained are compared in Table 1 and described individually below:

**Table 1.** Exposures estimated using ART 1.5, ACGIH exposure limits, and values reported in the PGR consulted, according to chemical substance

Chemical substance	Laboratory	Exposure estimated using ART 1.5	ACGIH limit (2021), TWA	ACGIH limit (2021), STEL	Value reported in the PGR consulted
Toluene	1	2.92 ppm	20 ppm	—	0.256 ppm
Acetone	1	6.37 ppm	250 ppm	500 ppm	7.642 ppm
Xylene	1	3.71 ppm	100 ppm	150 ppm	—
Benzene	1	0.0048 mg/m^3^ 0.1091 ppm	0.5 ppm	2.5 ppm	—
Gasoline	1	18 mg/m^3^ 4.78 ppm	300 ppm	500 ppm	—
Ethanol	1	0.00047 mg/m^3^ 0.0107 ppm	—	1,000 ppm	11.73 ppm
Methanol	1	0.0000057 mg/m^3^ 0.00013 ppm	200 ppm	250 ppm	—
Diesel fuel	1	0.00039 mg/m^3^ 0.0089 ppm	100 mg/m^3^	—	—
Diesel fuel	2	0.005 mg/m^3^ 0.1136 ppm	100 mg/m^3^	—	—

Source: Prepared by the authors based on estimates obtained using ART 1.5, the 2021 ACGIH threshold limit values, and the information consulted in the institutional PGR.

ACGIH = American Conference of Governmental Industrial Hygienists; ART 1.5 = Advanced REACH Tool, version 1.5; PGR = Risk Management Program; STEL = short-term exposure limit; TWA = time-weighted average. The dash (—) indicates that no limit was established by the ACGIH or that the information was not identified in the PGR, according to the corresponding column.

In laboratory 1, the 75th-percentile acetone concentration estimated for the worker’s breathing zone outside the respiratory protective equipment (RPE) was 24 mg/m^3^ (6.37 ppm), with an interquartile confidence interval of 12-48 mg/m^3^.

The estimated toluene concentration was 11 mg/m^3^ (2.92 ppm), with an interquartile confidence interval of 5.6-22 mg/m^3^.

The estimated xylene concentration was 14 mg/m^3^ (3.71 ppm), with an interquartile confidence interval of 7.3-29 mg/m^3^.

The estimated benzene concentration was 0.0048 mg/m^3^ (0.1091 ppm), with an interquartile confidence interval of 0.0024-0.0094 mg/m^3^.

The estimated methanol concentration was 0.0000057 mg/m^3^ (0.00013 ppm), with an interquartile confidence interval of 0.0000029-0.000011 mg/m^3^.

The estimated gasoline concentration was 18 mg/m^3^ (4.78 ppm), with an interquartile confidence interval of 9-35 mg/m^3^.

The estimated ethanol concentration was 0.00047 mg/m^3^ (0.0107 ppm), with an interquartile confidence interval of 0.00022-0.001 mg/m^3^.

The estimated diesel fuel concentration was 0.00039 mg/m^3^ (0.0089 ppm), with an interquartile confidence interval of 0.00019-0.00083 mg/m^3^.

In laboratory 2, the 75th-percentile diesel fuel concentration estimated for the worker’s breathing zone outside the RPE was 0.005 mg/m^3^ (0.1136 ppm), with an interquartile confidence interval of 0.0024-0.011 mg/m^3^.

## DISCUSSION

Exposure estimates were calculated based on the average daily duration of substance use, adopting a conservative assumption to represent a scenario of greater potential exposure. Even under these conditions, the concentrations estimated using ART 1.5 remained considerably below the time-weighted average occupational exposure limits established by the American Conference of Governmental Industrial Hygienists (ACGIH). Although the estimates also remained below the short-term exposure limit (STEL), this comparison should be interpreted with caution because the STEL applies to short-duration exposures and requires estimates corresponding to the same reference period.

However, the use of a predictive model entails inherent limitations because such tools are based on standardized scenarios and may not capture microenvironmental variations, intermittent exposures, or transient peaks.

In this context, direct quantitative monitoring is advisable, particularly in laboratory 2, which has limited ventilation and no local exhaust systems. Although the volume of samples handled is small, the combination of limited physical space and insufficient air exchange may affect sensory perception and occupational comfort, aspects not fully addressed by exposure limits.

Based on the documentary information accessed during the supervised consultation, weaknesses were identified in the characterization of chemical risks. The PGR materials consulted predominantly recorded agents such as acetone, cyclohexane, organic solvents and acids, ethanol, methyl ethyl ketone, carbon monoxide, and toluene in qualitative terms, without specifically quantifying some of the substances evaluated in this study, such as toluene, acetone, xylene, methanol, and diesel fuel. Carbon monoxide was also listed, although the corresponding source process could not be identified in the materials accessed. Furthermore, the generic categorization of “organic solvents and acids” lacked technical specificity, which may compromise the accuracy of the hazard inventory and the effectiveness of the proposed control measures.

A possible inconsistency was also observed between the information accessed in the PGR and PCMSO regarding benzene biomonitoring. According to the information consulted, trans, trans-muconic acid measurement was included in pre-employment and periodic examinations; however, no prior quantitative characterization of benzene as an occupational risk was identified in the PGR materials accessed. Because the documentary review was conducted through supervised and partial access to institutional documents, this finding should be interpreted with caution. Nevertheless, the observed disconnect between environmental hazard identification and biological surveillance suggests a possible methodological misalignment between the management instruments, reinforcing the need for an integrated review of the PGR and PCMSO.

Regarding structural safety, the absence of adequate escape route signage and formally designated emergency exits, in violation of NR-23, represents a significant vulnerability. Although alarm systems, hydrants, and periodic training are available, the absence of clearly established routes may compromise evacuation in critical situations, demonstrating that risk management should simultaneously address chemical and structural aspects.

Because this was a cross-sectional study, exposures and outcomes were assessed at a single point in time, limiting the analysis of temporality and precluding causal inferences. The small sample size (n = 12) limits the precision of the descriptive estimates and the generalizability of the findings. Signs and symptoms were self-reported, which may introduce information bias, including underreporting or overestimation of symptoms and difficulty attributing them to occupational exposure. The absence of direct environmental monitoring limits confirmation of actual workplace concentrations, particularly in microenvironments, during short-duration tasks, and in situations involving transient exposure peaks. Although ART 1.5 is useful for preliminary estimation, it is a predictive model based on scenarios and assumptions and may not fully capture microenvironmental variations, fluctuations in ventilation, and intermittent exposures. Furthermore, supervised and partial access to the PGR and PCMSO, without the complete files being made available to the researchers, limits the scope of the documentary assessment and warrants caution when interpreting the inconsistencies identified. Nevertheless, the findings contribute to an integrated understanding of the risks and reinforce the importance of coordinating exposure modeling, direct environmental monitoring, health surveillance, and the alignment of occupational management instruments.

## CONCLUSIONS

Under the modeled conditions, the exposures estimated using ART 1.5 for the agents evaluated remained below the occupational exposure limits established by the ACGIH. Compared with the values reported in the institutional PGR, the estimates were lower for acetone and ethanol and higher for toluene. Because ART 1.5 is a predictive tool based on specific input data and assumptions, the results should be interpreted as preliminary estimates and do not replace direct environmental monitoring.

Opportunities to improve the management instruments were identified, particularly regarding the quantitative characterization of chemical agents and the integration of the information accessed in the PGR and PCMSO. The need for structural improvements related to ventilation and escape route signage was also highlighted.

Although limited by its cross-sectional design, small sample size, use of predictive modeling, and partial access to institutional documents, the study contributes to the understanding of the risks present in fuel analysis laboratories and reinforces the importance of integrating environmental assessment, health surveillance, and preventive management.

## Data Availability

Upon publication, the data will be made available by the authors upon request.
